# CT and MR for bone mineral density and trabecular bone score assessment in osteoporosis evaluation

**DOI:** 10.1038/s41598-023-43850-z

**Published:** 2023-10-03

**Authors:** Haein Lee, Sunghoon Park, Kyu-Sung Kwack, Jae Sung Yun

**Affiliations:** 1 Department of Radiology, Ajou University School of Medicine, 164, World Cup-Ro, Yeongtong-Gu, Suwon, 16499 South Korea; 2 Musculoskeletal Imaging Laboratory, Ajou University Medical Center, Suwon, South Korea

**Keywords:** Diseases, Health care, Medical research

## Abstract

Dual energy X-ray absorptiometry (DXA) is widely used modality for measuring bone mineral density (BMD). DXA is used to measure the quantitative areal BMD of bone, but has the disadvantage of not reflecting the bone architecture. To compensate for this disadvantage, trabecular bone score (TBS), a qualitative parameter of trabecular microarchitecture, is used. Meanwhile, there have been recent attempts to diagnose osteoporosis using the Hounsfield unit (HU) from CT and MR-based proton density fat fraction (PDFF) measurements. In our study, we aimed to find out the correlation between HU/PDFF and BMD/TBS, and whether osteoporosis can be diagnosed through HU/PDFF. Our study revealed that the HU value showed a moderate to good positive correlation with BMD and TBS. PDFF showed a fair negative correlation with BMD and TBS. In diagnosing osteopenia and osteoporosis, the HU value showed good performance, whereas the PDFF showed fair performance. In conclusion, both HU values and PDFF can play a role in predicting BMD and TBS. Both HU values and PDFF can be used to predict osteoporosis; further, CT is expected to show better results.

## Introduction

Osteoporosis is a systemic skeletal disease characterized by low bone mass and microarchitectural deterioration of bone tissue^1^. Osteoporosis has been classified into two categories, primary and secondary, according to the presence or absence of a causal disease^2, 3^. Primary osteoporosis is further divided into postmenopausal osteoporosis (type I) and senile osteoporosis (type II). Type I osteoporosis develops in women who have an estrogen deficiency, leading to a predominant loss of trabecular bone as opposed to cortical bone. Type II osteoporosis occurs in men and women through the loss of stem-cell precursors, with a predominant loss of cortical bone.

Currently, dual-energy X-ray absorptiometry (DXA) is recommended by the World Health Organization (WHO) as the gold standard technique and most widely used modality for measuring bone mineral density (BMD), which is a quantitative measurement of bone used to predict bone strength and fracture risk^4^. However, due to its projectional nature, DXA cannot differentiate trabecular from cortical BMD^5^. Given the limitations of DXA, researchers have investigated tools to assess not just density but bone architecture. Thus, DXA images of the lumbar spine have been used to evaluate pixel gray-level texture to define a trabecular bone score (TBS), which is an indirect index and qualitative parameter of trabecular microarchitecture^4–6^. In theory, a dense trabecular network, correlated with higher mechanical bone strength, generates a projection image characterized by numerous small-amplitude gray-level texture variations. Consequently, this leads to a steep variogram slope and a high TBS value, associated with better bone structure. Conversely, a low TBS value reflects a reduced number of gray-level texture variations with larger amplitudes, resulting in a shallower slope and indicative of worse bone structure. Several studies have shown that low TBS is associated with an increased prevalence of fragility fractures in postmenopausal women and in older men^7–9^.

Quantitative computed tomography (QCT) has some advantages over DXA in diagnosing osteoporosis. Most importantly, QCT allows true volumetric measurements of the lumbar spine independent of body size that discriminates trabecular and cortical compartments^5^. Furthermore, QCT is known to be more sensitive than DXA for the treatment of osteoporosis and advantageous over DXA in cases of rheumatological disorders such as ankylosing spondylitis. Nevertheless, QCT has some limitations as a primary diagnostic tool for osteoporosis due to its relatively high cost and greater amount of radiation exposure^10, 11^. To overcome these limitations, opportunistic screening for osteoporosis using the Hounsfield unit (HU) from CT has recently been attempted^12–14^. The HU value is positively correlated with material density and compressive strength^15, 16^. Opportunistic screening has the advantages of not requiring additional imaging, radiation exposure, or appointments^12, 13^.

Recently, there have been attempts to diagnose osteoporosis using MR-based proton density fat fraction (PDFF) measurements^17, 18^. In the bone marrow, osteoblasts and adipocytes differentiate from common progenitor cells^19^. In osteoporosis, there is a predominance of differentiation toward adipocytes in progenitor cells, while differentiation toward osteoblasts decreases, leading to reduced bone formation. As a result, the increase in bone marrow fat becomes an indirect indicator of osteoporosis and a target for osteoporosis diagnosis. MR-based PDFF measurements are a reliable tool for evaluating vertebral bone marrow fat quantification without radiation exposure^20, 21^.

While there have been attempts to measure BMD with various tools, there have been no attempts to reveal a relationship with TBS. We hypothesized that HU or PDFF values may be related to BMD or TBS. In this study, we compared whether BMD and TBS differ according to sex and age. And we evaluated the relationship between the HU/PDFF values and BMD/TBS. In addition, the diagnostic performance of HU/PDFF values in predicting osteopenia and osteoporosis was analyzed.

## Results

### Patient cohort

**Figure 1:**
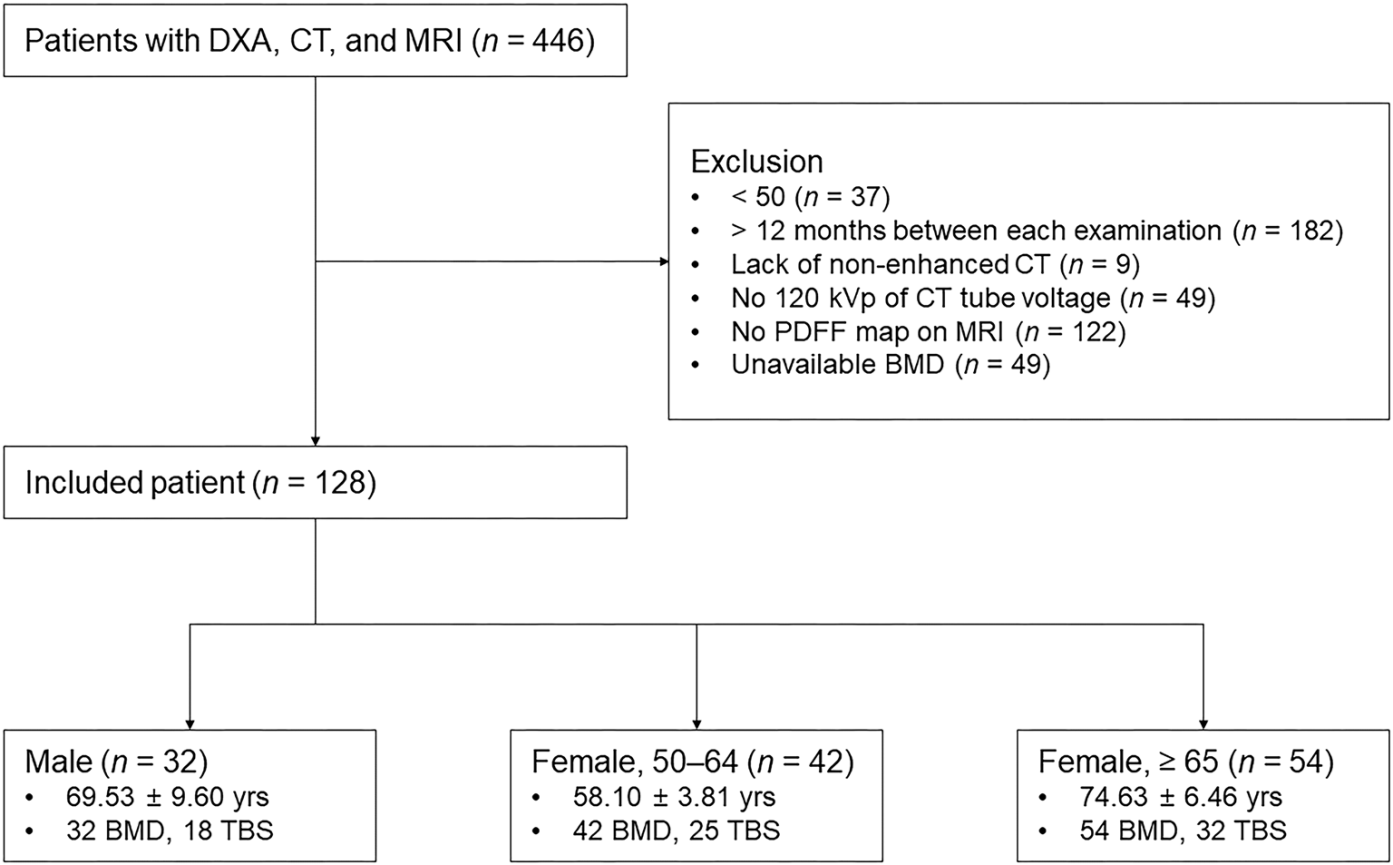
Flowchart showing selection criteria and patient characteristics. Patients were divided into 3 groups according to age and sex. DXA—dual-energy X-ray absorptiometry, PDFF—proton density fat fraction, BMD—bone mineral density, TBS—trabecular bone score.

Patients were grouped into males and females aged 50–64 years, and females aged ≥ 65 years. The number of patients in each group was 32, 42, and 54, respectively (Fig. 1). The average age of males and females was 69.5 ± 9.6 and 67.4 ± 9.9, respectively, and there was no statistically significant difference (*p* = 0.288). The examination interval between DXA-CT was 2.3 months, and the interval between BMD–MR and CT–MR was 2.1 months, respectively. Patients were diagnosed with osteopenia, osteoporosis, or as normal according to the T-score of DXA. In total, 50 and 35 patients were diagnosed with osteoporosis and osteopenia, respectively, and 43 patients were classified as normal.

### Interobserver and intraobserver agreement

Interobserver agreements between the two radiologists for mean HU values and PDFF were excellent, with intraclass correlation coefficient (ICC) values of 0.983 (95% confidence interval [CI], 0.976–0.988) and 0.994 (95% CI, 0.991–0.995), respectively. Intraobserver agreements were also excellent. The ICC values for mean HU values and PDFF were 0.991 (95% CI: 0.987–0.993) and 0.990 (0.984–0.993), respectively.

### Comparisons of BMD, TBS, HU and PDFF by gender and age

The average BMD in males and females was 1.059 ± 0.203 g/cm^2^ and 0.905 ± 0.172 g/cm^2^, respectively. The difference in BMD between the two groups was statistically significant (*p* < 0.001). However, there was no statistically significant difference in TBS between the two groups. (1.409 ± 0.104 vs. 1.360 ± 0.101, *p* = 0.08). HU values and PDFF also showed no statistically significant difference (Table 1).

**Table 1:** Comparison of males and females, and subgroups of females.

	HU		PDFF	
	Mean ± SD	*p*	Mean ± SD	*p*
Male	106.75 ± 41.35	0.102	59.58 ± 9.88	0.779
Female	92.61 ± 42.27		60.09 ± 8.64	
Age 50–64	118.73 ± 39.34	< 0.001	57.83 ± 8.23	0.023
Age > 65	72.30 ± 32.31		61.86 ± 8.62	

*HU* Hounsfield unit; *PDFF* proton density fat fraction; *SD* standard deviation.

The average BMD in female subgroups was significantly lower in the older group, measuring 0.957 ± 0.168 g/cm^2^ in the age 50–64 group and 0.865 ± 0.165 g/cm^2^ in the age ≥ 65 group (*p* = 0.008). The TBS was also significantly different in females according to age (1.398 ± 0.096 vs. 1.330 ± 0.095, *p* = 0.01). HU values and PDFF between the two groups also showed statistically significant differences (Table 1).

### Correlations between age and BMD/TBS

In females, there was a fair negative correlation between BMD and TBS according to age (Table 2). In particular, in the age 50–64 female group, TBS showed a moderate to good correlation according to age (*r* =  − 0.521). However, neither BMD nor TBS showed a statistically significant correlation with age in males or in females aged over 65 years.

**Table 2:** Correlations between age and BMD/TBS.

	BMD		TBS	
	*r*	*P*	*r*	*p*
Male	− 0.037	0.840	− 0.377	0.123
Female	− 0.283	0.005	− 0.395	0.002
Age 50–64	− 0.175	0.268	− 0.521	0.008
Age > 65	− 0.085	0.543	− 0.048	0.792
All	− 0.167	0.060	− 0.325	0.005

*BMD* bone mineral density; *TBS* trabecular bone score.

### Correlations between HU/PDFF and BMD/TBS

The HU value showed a moderate to good positive correlation with BMD and TBS (*r* = 0.693 and 0.528, respectively). In subgroup analysis, TBS correlated better with the male group (Table 3).

**Table 3:** Correlations between HU/PDFF and BMD/TBS.

	BMD				TBS			
	HU		PDFF		HU		PDFF	
	*r*	*p*	*r*	*p*	*r*	*p*	*r*	*p*
Male	0.708	< 0.001	− 0.373	0.035	0.693	0.001	− 0.663	0.003
Female	0.691	< 0.001	− 0.316	0.002	0.460	< 0.001	− 0.245	0.067
Age 50–64	0.664	< 0.001	− 0.208	0.186	0.458	0.021	0.012	0.955
Age > 65	0.689	< 0.001	− 0.317	0.019	0.261	0.149	− 0.317	0.077
All	0.693	< 0.001	− 0.321	< 0.001	0.528	< 0.001	− 0.307	0.008

*HU* Hounsfield unit; *PDFF* proton density fat fraction; *BMD* bone mineral density; *TBS* trabecular bone score.

PDFF showed a fair negative correlation with BMD and TBS (*r* =  − 0.321 and − 0.307, respectively). In subgroup analysis, PDFF showed a fair negative correlation with BMD in both males and females (*r* =  − 0.373 and − 0.316, respectively). However, PDFF values and TBS showed a moderate to good correlation only within the male group (*r* =  − 0.663), while no correlation was observed within the female group (*p* = 0.067) (Table 3).

### Comparisons of HU/PDFF by normal bone density/osteopenia/osteoporosis

The differences in mean HU values between normal, osteopenia, and osteoporosis groups were significant (Table 4). The mean HU values from normal, osteopenia, and osteoporosis subjects, respectively, were 135.46, 91.43, and 65.64.

**Table 4:** Comparison of normal and osteopenia groups, and osteopenia and osteoporosis groups.

Normal versus Osteopenia	
HU	
Normal	135.46 ± 35.67
Osteopenia	91.43 ± 24.75
*p*	< 0.001
PDFF	
Normal	56.38 ± 7.67
Osteopenia	59.04 ± 8.93
*p*	0.161
Osteopenia versus Osteoporosis	
HU	
Osteopenia	91.43 ± 24.75
Osteoporosis	65.64 ± 28.36
*p*	< 0.001
PDFF	
Osteopenia	59.04 ± 8.93
Osteoporosis	63.81 ± 8.65
*p*	0.018

*HU* Hounsfield unit, *PDFF* proton density fat fraction.

The PDFF of the osteoporosis group was significantly higher than the PDFF of the osteopenia and normal groups (Table 4), while the difference between the normal and osteopenia group was not significant.

### Diagnostic performance of CT and MR for predicting osteopenia and osteoporosis

**Figure 2:**
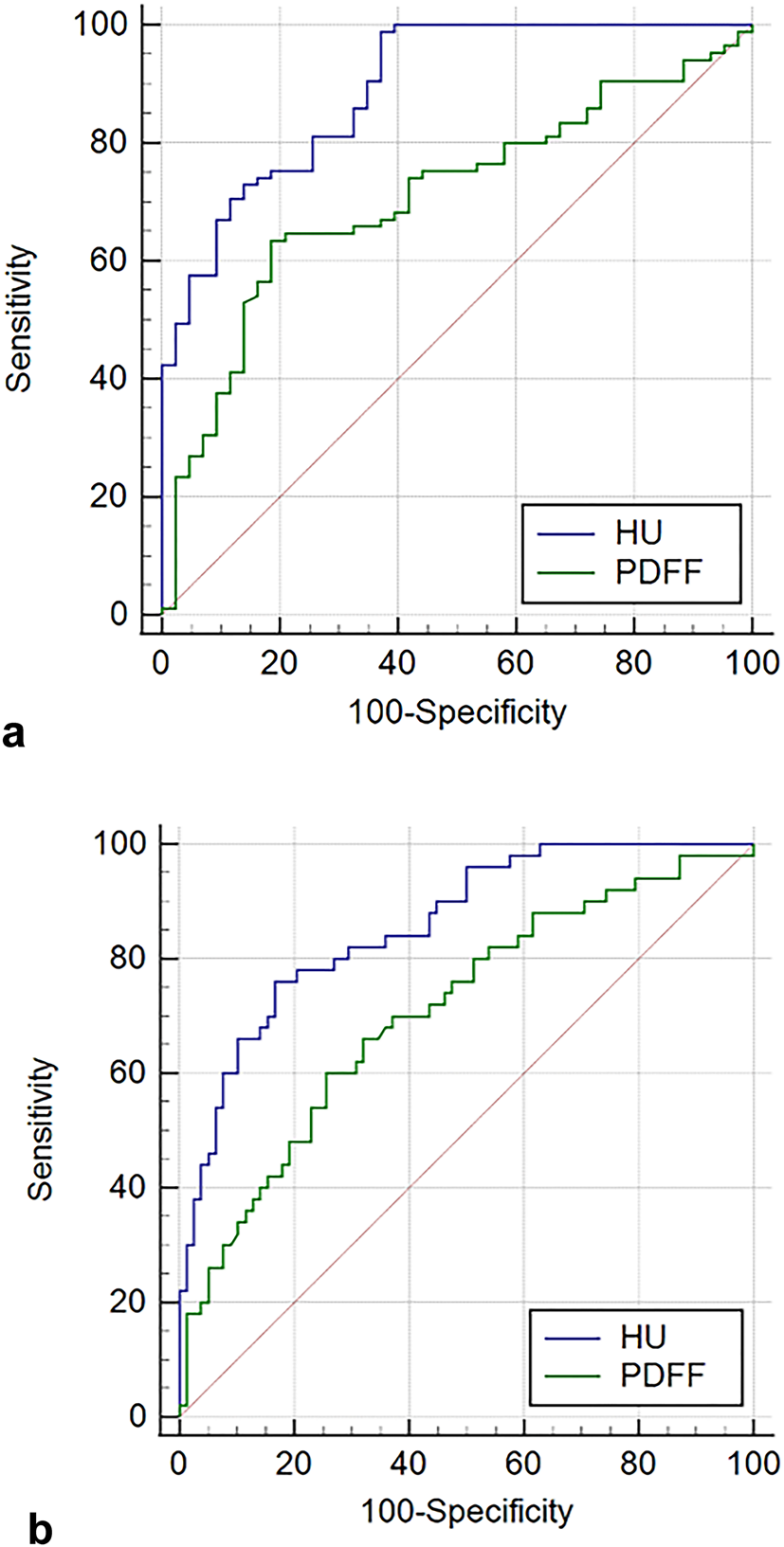
Graphs showing the ROC curves for predicting osteopenia and osteoporosis. (**a**) Distinguishing between normal and osteopenia/osteoporosis, HU values showed good performance in both readers (AUC = 0.892). PDFF showed fair performance (AUC = 0.709). (**b**) In predicting osteoporosis, ROC analysis showed that the HU values showed good performance (AUC = 0.859). Fair performance was found in PDFF (AUCs = 0.707). ROC—receiver operating characteristic, HU—Hounsfield unit, AUC—area under curve, PDFF—proton density fat fraction.

For differentiating normal and osteopenia/osteoporosis subjects, HU values showed good performance (Fig. 2a). The area under curves (AUCs) was 0.892 and the cutoff values were 125.6. The AUCs and cutoff values of PDFF were 0.709 and 61.4, respectively (Table 5).

**Table 5:** Diagnostic performance of HU and PDFF in predicting osteopenia and osteoporosis.

	Normal versus Osteopenia		Osteopenia versus Osteoporosis		Normal versus Osteopenia and Osteoporosis		Normal and Osteopenia versus Osteoporosis	
	HU	PDFF	HU	PDFF	HU	PDFF	HU	PDFF
AUC	0.831	0.632	0.765	0.638	0.892	0.709	0.859	0.707
Sensitivity (%)	100.00	57.14	66.00	88.00	98.82	63.53	76.00	60.00
Specificity (%)	60.47	81.40	80.00	34.29	62.79	81.40	83.33	74.36
Cutoff value	128.2	61.36	69.51	55.33	125.63	61.36	75.43	62.77
*p*	< 0.001	0.050	< 0.001	0.023	< 0.001	< 0.001	< 0.001	< 0.001

*HU* Hounsfield unit, *PDFF* proton density fat fraction, *AUC* area under curve.

Better diagnostic performance in terms of differentiating normal/osteopenia and osteoporosis was achieved by HU values (AUC = 0.859) rather than PDFF (AUC = 0.707) (Fig. 2b). The HU cutoff values were 75.4 with a sensitivity of 76.0% and a specificity of 83.3% (Table 5). The PDFF cutoff values for osteoporosis were 62.8 with a sensitivity of 60.0% and a specificity of 74.4%.

## Discussion

This study was conducted to investigate the role of CT and MR in predicting BMD and TBS. Previously, several studies tried to diagnose osteoporosis through CT or MR^12–14, 17, 18^, but to the best of our knowledge, this study is the first to reveal a relationship with TBS. In this study, the HU value from CT and the PDFF from MR showed a correlation with BMD and TBS. It was found that these associations showed differences according to sex and age. Both HU and PDFF showed excellent reproducibility and could be used for the diagnosis of osteoporosis.

Areal BMD using DXA has an advantage insofar as the WHO criteria can be applied to the diagnosis of osteoporosis^22^. However, DXA has several limitations, and to overcome this, opportunistic screening using CT has been studied^12–14^. For accurate measurement, some studies have performed calibration using a phantom^23, 24^. However, calibration phantoms are expensive and are not always available. On the other hand, HU values can be easily measured, so they can be conveniently used even where a phantom is not prepared. In the same manner, PDFF can also be easily measured in  picture archiving and communication system (PACS), so it can be conveniently used for screening osteoporosis. As it showed excellent interobserver and intraobserver agreement in HU/PDFF measurement, this suggests there is no issue in terms of precision.

We divided patients into three groups: males, females aged 50–64 years, and females aged ≥ 65 years. We assumed females aged 50–64 years were susceptible to type I osteoporosis and females aged ≥ 65 years were susceptible to type II osteoporosis according to indications for BMD testing of the International Society for Clinical Densitometry (ISCD) ^22^. BMD was significantly different for males and females, and also between the female subgroups. The result of a lower BMD in females, especially in older people, was in good agreement with the result of a previous study^25^. On the other hand, there was no statistically significant difference in TBS between males and females (*p* = 0.078). This may be due to the fact that the number of males for whom TBS was measured was very small (*n* = 18) and the mean age was relatively high (male vs. female, 71.5 ± 9.8 vs. 66.5 ± 9.0, *p* = 0.046), so there may have been a selection bias. In comparison between the female subgroups, the older group showed significantly lower BMD and TBS results, which was consistent with previous studies^25, 26^. In the correlation between age and BMD/TBS, TBS was better in females aged 50–64 than in the other group, which is thought to reflect the predominant trabecular bone loss in type I osteoporosis.

Our study showed that BMD and HU values had a moderate to good correlation in all groups. However, depending on the subgroup, PDFF showed a fair to no correlation with BMD. Although there is theoretical background information that bone mass decreases in osteoporosis and adipose tissue accumulation increases instead^27^, it suggests that the HU value is superior to the PDFF in diagnosing osteoporosis. Similarly, the correlation between HU value and TBS was higher than that of the PDFF. Since DXA and CT are examinations that are performed on the same principle using radiation, the correlation appears to be higher than that of MR. Interestingly, the correlation with TBS was higher in both HU values and PDFF in the male group. In postmenopausal females, trabecular bone is relatively decreased due to the decrease in estrogen. This inhomogeneous bone marrow state seems to induce a weak correlation with HU/PDFF values.

HU values had a good diagnostic performance in differentiating between normal, osteopenia, and osteoporosis. The mean HU value of each group was 135.46, 91.43, and 65.64, respectively. Our study shows a relatively lower mean HU value in all three groups compared to previous studies^15, 28^. This inconsistency with our result may be due to the older age of our subjects. Previous studies included younger patients. However, this study only included patients over 50 years of age. Considering that screening for primary osteoporosis is generally performed in the elderly, the results of this study may fit the reality better.

The PDFF for the osteoporosis group was significantly higher than for the osteopenia and normal groups for both readers. However, our data showed no significant difference in PDFF between the normal and osteopenia/osteoporosis groups. This indicates that PDFF have limitations in replacing the DXA examination. Although receiver operating characteristic (ROC) analysis was able to diagnose normal bone density, osteopenia, and osteoporosis using PDFF, it is questionable whether it will actually be useful. The cutoff value for PDFF between the normal and osteopenia/osteoporosis groups was 61.36, and between osteoporosis and the normal/osteopenia groups, it was 62.77, with a difference of only 1.41. Considering that the standard deviation of PDFF ranged from 8.23 to 9.88 depending on gender/age, it appears to be a very small value. Comparing the cutoff value difference of 64.27 and the standard deviation range of 32.31–42.27 for HU, it is evident that there are clear limitations in using PDFF to diagnose osteoporosis.

Our study has several limitations. First, the number of patients included in this study was small. Due to the small sample size, it may be difficult to generalize our research results. Second, our study was a retrospective single-center study, and a selection bias may not have been avoidable. If a large number of patients are recruited prospectively for a future study, it may reduce selection bias. Third, various CT machines and protocols were used. Although there was a report that there was no significant difference in HU measurement according to the scanner^29^, and daily calibration was performed, the diversity of CT machines and protocols may limit the generalizability of our results. Fourth, only 75 among 128 patients had TBS data. Fifth, we included women over 50 who were not actual postmenopausal women for type I osteoporosis.

In conclusion, TBS reflected trabecular bone loss in type I osteoporosis. The HU value of CT showed a moderate to good correlation with BMD and TBS, demonstrating a stronger correlation compared to the fair correlation observed with PDFF. In predicting TBS, the application of HU values and PDFF for males is more advantageous than for females. Both CT and MR are available for predicting osteoporosis, but CT showed superior results over MR. This study raises expectations that CT and MR, particularly CT, may play a role in screening for osteoporosis.

## Materials and methods

### Patient selection

This retrospective study was approved by the Institutional Review Board of Ajou University Hospital (IRB no. AJOUIRB-MDB-2022–107), and the requirement for informed consent was waived. All methods in the present study were performed in accordance with the relevant guidelines and regulations. From January 2017 to December 2021, a total of 466 patients who received DXA, CT, and MR of the lumbar spine were enrolled. We evaluated all CT examinations of the lumbar spine region, including lumbar spine CT, abdominal CT, and vascular CT. Exclusion criteria were as follows: (1) younger than 50 years; (2) over a 12 month time interval between each examination; (3) lack of non-enhanced CT; (4) not 120 kVp of CT tube voltage; (5) lack of PDFF map on MR; and (6) unavailable BMD due to structural abnormalities such as fracture, deformity, tumor, infection, or previous operation. The final study population consisted of 128 patients (32 males and 96 females; mean age 67.9 ± 9.8 years; age range 50–91 years). Among those who underwent DXA, the TBS was measured for 75 patients. Patients were divided into three subgroups: males, females aged 50–64 years, and females aged ≥ 65 years (Fig. 1).

### Dual-energy X-ray absorptiometry

All DXA examinations of the lumbar spine (L1–4) were performed using a Lunar Prodigy scanner (GE Healthcare, Madison, WI, USA). For each vertebra, the manufacturer’s software automatically calculated areal BMD (g/cm^2^) and T-scores. BMD measurements were made in at least two vertebrae according to ISCD recommendations^11, 22, 30^. BMD should gradually increase from L1 to L4, and each vertebra should be within 1 standard deviation of the next. The TBS was also obtained from the vertebra at the selected vertebra for BMD measurement. Mean values were obtained from BMD, T-score, and TBS obtained from each vertebra. To ensure consistency in the measurement locations of BMD and TBS, BMD from the hip was not utilized. Normal BMD, osteopenia, and osteoporosis were defined as T-score ≥  − 1.0, − 2.5– − 1.0, and ≤  − 2.5, respectively.

### Computed tomography

CT was performed using seven multidetector CT scanners (Siemens Somatom Definition Edge, Definition Flash, and Force, Siemens Healthineers, Erlangen, Germany; Philips Brilliance 64 and Brilliance 16, Philips Healthcare, Best, Netherlands; Canon Aquilion One, Canon Medical Systems, Otawara, Japan; GE Revolution EVO, Madison, WI, GE Healthcare). CT was performed using a peak tube voltage of 120 kVp, a slice thickness of 2.0–5.0 mm, and adaptive tube current in helical mode. CT scans were performed using a bone kernel or a soft tissue kernel depending on the imaging purpose. All CT machines were calibrated daily with external air. The HU value of air should remain relatively constant, but it can vary slightly depending on factors such as temperature and humidity. Therefore, a calibration process is performed by conducting a scan using only air, without any specific object or phantom inside the CT bore, to adjust the HU value. Two-dimensional reconstructions were acquired in the coronal and/or sagittal planes with a bone or soft tissue kernel and a thickness of 2.0–5.0 mm.

### Magnetic resonance imaging

Spinal MR was performed using a 3-T MR scanner (Discovery MR750w, GE Healthcare, Madison, WI, GE Healthcare and Ingenia Elition X, Philips Healthcare, Best, Netherlands). Conventional MR sequences of the lumbar spine included at least a sagittal T1-weighted spin-echo, sagittal fat-suppressed T2-weighted spin-echo, axial T1-weighted spin-echo, and axial T2-weighted spin-echo images. Field of view, matrix, size, slice thickness, and interslice gap were tailored to the specific site under study. Detailed imaging parameters are summarized in Supplementary Table S1 online.

For PDFF estimation, six-echo 3D gradient-echo modified chemical shift-encoded images (IDEAL-IQ, GE Healthcare and mDixon Quant, Philips Healthcare) were acquired. The imaging parameters are summarized in Supplementary Table S2 online. Following acquisition, each image was reconstructed automatically and simultaneously into PDFF maps.

### Image analysis

**Figure 3:**
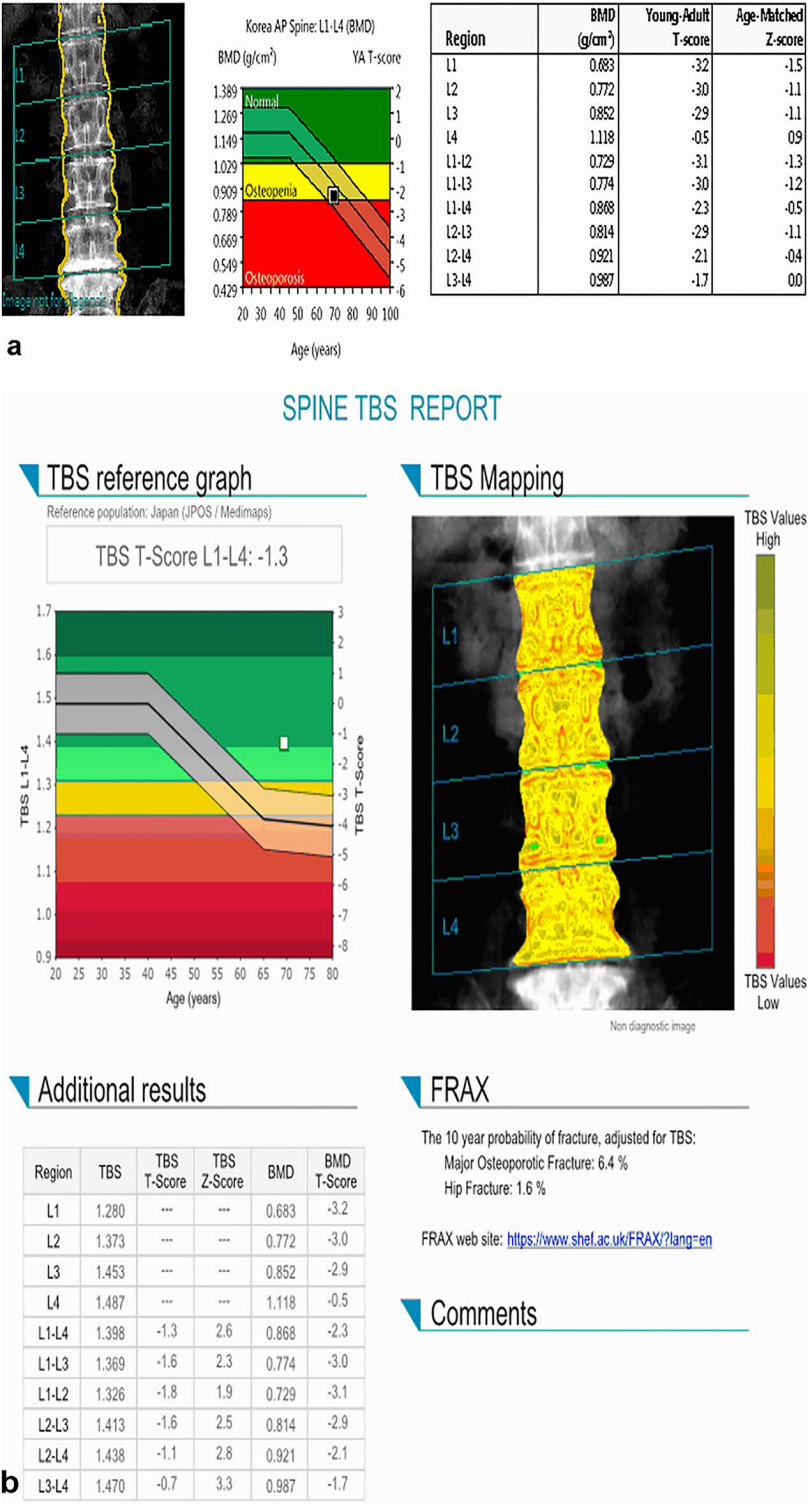

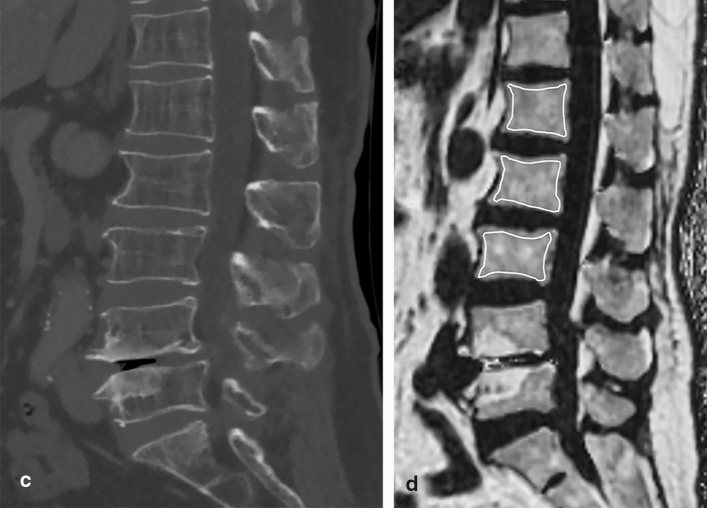
DXA scan, CT scan, and MR PDFF map of a 69-year-old female patient. (**a**) The T-score of L1-4 in the DXA scan is − 3.2, − 3.0, − 2.9, and − 0.5, respectively. Since the T-score of L4 increased by more than 1 SD than that of L3, it is excluded from the measurement. Therefore, the BMD of this patient is measured to be 0.774 g/cm^2^, which is the mean of L1–3. (**b**) TBS is measured as 1.369 as the average value of L1–3 selected for BMD measurement. (**c**) A sagittal CT image show sclerotic changes around the lower endplate of L4. HU measurements are performed in L1–3, and the mean HU values are 54.71 (**d**) PDFF of L1–3 bodies are measured in the MR PDFF map, showing the region of interest to measure PDFF in each body. PDFF are 62.98. DXA—dual-energy X-ray absorptiometry, PDFF—proton density fat fraction, SD—standard deviation, BMD—bone mineral density, TBS—trabecular bone score, HU—Hounsfield unit.

HU values and PDFF were measured independently by two radiologists blinded to the patients’ DXA scores. ROIs were manually drawn on the selected vertebral bodies used for BMD measurements in DXA. In CT, the largest possible elliptical ROIs were drawn to avoid the cortical margin at three different levels of the axial images of each bone (Supplementary Figure S1 online)^12, 31^. Mean HU values were calculated for the ROIs using the INFINITT PACS. An average of the 3 measurements determined the HU for individual vertebral levels. The ROIs included only cancellous bone, and cortical edges, osseous abnormalities, and venous plexus were avoided. In MR, ROIs were measured on the sagittal slice at the central region of the vertebral body. Manual ROIs were drawn along the outer border of the vertebral body to encompass as much of the bone marrow area as possible while avoiding confounding structures, such as the bony cortex and vertebral venous plexus (Fig. 3)^32, 33^. The HU values and PDFF measured in the selected L1–4 bodies were averaged and used as a representative value for each individual.

One radiologist repeated his measurements after one month to assess intraobserver agreement.

### Statistical analysis

Interobserver and intraobserver agreement between HU and PDFF measurements was analyzed using the ICC value. Inter- and intraobserver agreement were classified as follows: < 0.50, poor; 0.50–0.75, moderate; 0.75–0.90, good; and > 0.90, excellent^34^. We compared BMD, TBS, HU, and PDFF values between males and females and female subgroups using an independent *t* test. In each subgroup, the correlation between age and BMD/TBS was obtained through Pearson’s correlation analysis (*r*). Pearson’s correlation analysis was also performed to analyze the correlation between mean HU/PDFF and BMD/TBS in all patients and subgroups. The degree of correlation was scored as follows: 0–0.25, little; 0.25–0.5, fair; 0.5–0.75, moderate to good; ≥ 0.75, very good to excellent^32^. An independent *t* test was conducted to compare mean HU values and PDFF of normal, osteopenia, and osteoporosis groups. To determine the diagnostic performance of the parameters for predicting osteopenia and osteoporosis, ROC curves were generated. The AUCs were calculated, and optimal mean HU values were obtained by each reader. AUCs were compared using the DeLong test and interpreted as follows: 0.90–1.0, excellent; 0.80–0.90, good; 0.70–0.80, fair; 0.60–0.70, poor; and 0.50–0.60, failed^17^. Statistical analyses were performed using commercially available software (MedCalc version 20.106; MedCalc Software, Ostend, Belgium). A *p* value < 0.05 was considered statistically significant.

### Supplementary Information

Supplementary Information.

## Data Availability

The datasets used and/or analysed during the current study available from the corresponding author on reasonable request.
