# Supplementary material for: CT and MR for bone mineral density and trabecular bone score assessment in osteoporosis evaluation
Source: Sci Rep. 2023 Oct 3;13:16574. doi: 10.1038/s41598-023-43850-z (PMC10547782; doi:10.1038/s41598-023-43850-z)
Supplement: Supplementary file 1 — Supplementary Information. [file 41598_2023_43850_MOESM1_ESM.pdf]

CT and MR for bone mineral density and trabecular bone score assessment in osteoporosis evaluation

Haein Lee[1,2], Sunghoon Park[1,2], Kyu-Sung Kwack[1,2], Jae Sung Yun[1,2*]

[1] Department of Radiology, Ajou University School of Medicine, Suwon, South Korea

[2] Musculoskeletal Imaging Laboratory, Ajou University Medical Center, Suwon, South Korea

Supplementary materials

Supplementary Table S1. Summary of MR parameters for conventional MR sequences

	Sagittal T1W	Sagittal FS T2W	Axial T1W	Axial T2W
Repetition time (ms)	450–850	2400–3500	490–800	3000–7000
Echo time (ms)	9–14	80–150	9–17	90–150
Matrix size	384 × 256–416 × 260	384 × 256–416 × 260	384 × 260	384 × 260
Field of view (cm)	280 × 280–330 × 330	280 × 280–330 × 330	240 × 240	240 × 240
Slice thickness (mm)	3.5	3.5	4	4
Intersection gap (mm)	0.35	0.35	0.4	0.4

T1W T1-weighted spin-echo, FS fat-suppressed, T2W T2-weighted spin-echo

Supplementary Table S2. Summary of MR parameters for fat quantification

	IDEAL-IQ	mDixon-Quant
Repetition time (ms)	8.5	7.6
Echo time (ms)	1.3 (TE 1), ΔTE = 1.06	1.26 (TE 1), ΔTE = 1.0
Matrix size	192 × 192	280 × 280
Field of view (cm)	33 × 30	28 × 28
Slice thickness (mm)	3.5	3.85
Intersection gap (mm)	0.35	0
Flip angle (°)	4	3
Acceleration factor	2	1
Scan time	1 min 53 sec	41 sec

Supplementary Figure S1

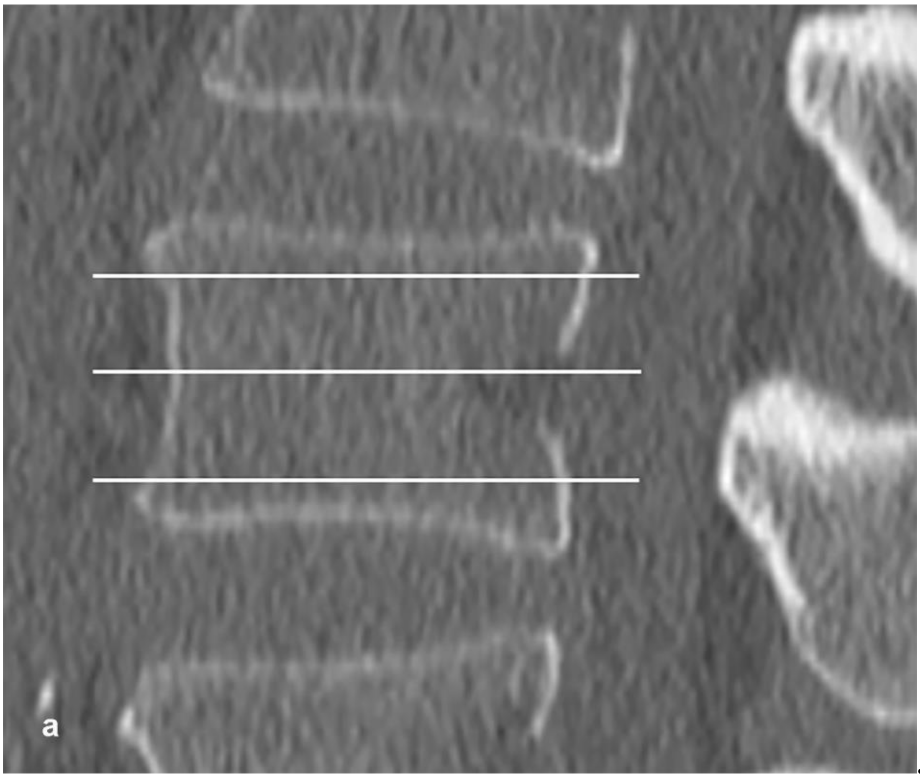

.

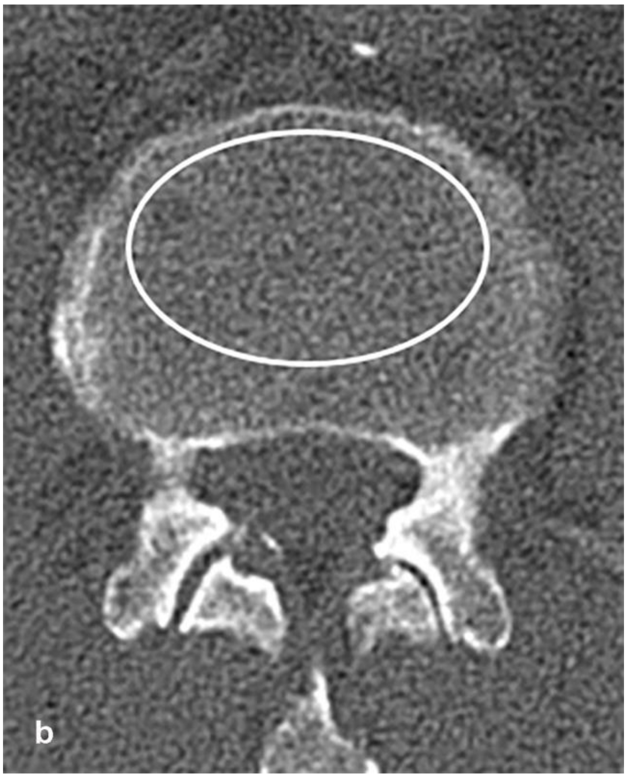

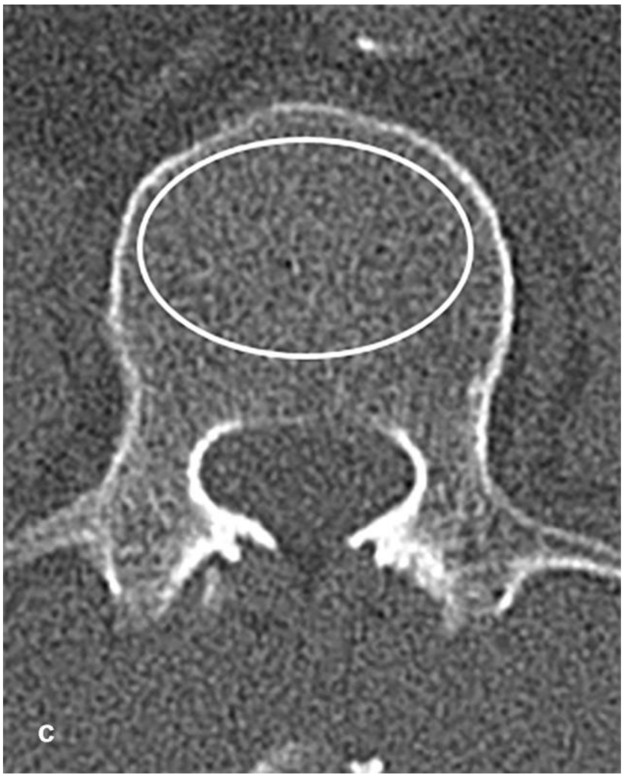

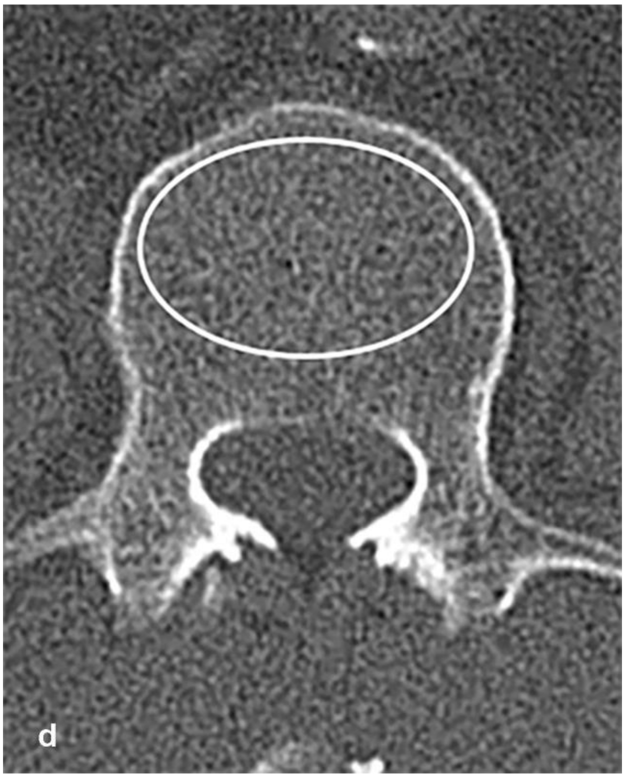

Supplementary Figure S1. CT scans show the technique to calculate vertebral BMD with HUs. **a.** Sagittal slice of the L2 vertebral body illustrate axial planes of interest. **b–d.** Axial images show axial planes with the placement of the region of interest. The mean HU value of

B–D is used for calculating vertebral body bone density. BMD bone mineral density, HU Hounsfield unit
